# From multi-omics integration towards novel genomic interaction networks to identify key cancer cell line characteristics

**DOI:** 10.1038/s41598-021-90047-3

**Published:** 2021-05-18

**Authors:** T. J. M. Kuijpers, J. C. S. Kleinjans, D. G. J. Jennen

**Affiliations:** Department of Toxicogenomics, GROW School for Oncology and Developmental Biology, Maastricht University, P.O. Box 616, 6200 MD Maastricht, the Netherlands

**Keywords:** Data integration, Network topology, Cancer epigenetics, Cancer genetics

## Abstract

Cancer is a complex disease where cancer cells express epigenetic and transcriptomic mechanisms to promote tumor initiation, progression, and survival. To extract relevant features from the 2019 Cancer Cell Line Encyclopedia (CCLE), a multi-layer nonnegative matrix factorization approach is used. We used relevant feature genes and DNA promoter regions to construct genomic interaction network to study gene–gene and gene—DNA promoter methylation relationships. Here, we identified a set of gene transcripts and methylated DNA promoter regions for different clusters, including one homogeneous lymphoid neoplasms cluster. In this cluster, we found different methylated transcription factors that affect transcriptional activation of EGFR and downstream interactions. Furthermore, the hippo-signaling pathway might not function properly because of DNA hypermethylation and low gene expression of both LATS2 and YAP1. Finally, we could identify a potential dysregulation of the CD28-CD86-CTLA4 axis. Characterizing the interaction of the epigenome and the transcriptome is vital for our understanding of cancer cell line behavior, not only for deepening insights into cancer-related processes but also for future disease treatment and drug development. Here we have identified potential candidates that characterize cancer cell lines, which give insight into the development and progression of cancers.

## Introduction

Different hallmarks of cancer have been identified that contribute to the development and propagation of tumors^1^. These hallmarks include sustaining proliferative signaling, evading growth suppressors, resisting cell death, and activating invasion and metastasis. Evading growth suppressors is achieved by the inhibition of the expression of certain genes, called tumor suppressor genes^1^. Tumor suppressor genes regulate important processes such as preventing unrestrained cellular growth, DNA repair promotion, and cell cycle checkpoint activation^2^. Besides tumor suppressor genes, oncogenes play a crucial role in regulating cellular growth, division, and survival^2^. Tumorigenesis is likely to be driven by events that result in the gain of an oncogene or the loss of the suppressor gene, and tumor maintenance often depends on continued oncogene activity^3^. However, the order in which both events happen differ per tumor type. Most hematopoietic cancers and soft-tissue sarcomas are initiated by oncogene activation, followed by alterations in tumor-suppressor genes and other oncogenes^4^. Whereas some carcinomas are initiated by first, a loss of function of a tumor-suppressor gene, and second, alterations in oncogenes and additional tumor-suppressor genes^4^. Although mutations in tumor suppressor genes are important, it is not the only mechanism responsible for alternated gene expression^5^. Genomic instability plays a major part in the activation of oncogenes and subsequently, the inhibition of tumor suppressor genes, thus suggesting a role for epigenomics. For example, inactivation of BRCA1 in sporadic breast cancer is not due to a mutation but promoter hypermethylation^6^.

Almost all cancer cells show genomic instability^7^. In healthy cells, chromatin and associated epigenetic mechanisms ensure stable gene expression and cellular states. Cancer cells show important alterations in these epigenetic mechanisms, which represent one of the fundamental characteristics of nearly all human cancers^8^. A large number of cancer cells show an increase in methylation of normally unmethylated CpG islands and promoter regions of tumor suppressors and DNA repair genes^9^. It has been shown that the increase in DNA methylation increases genomic instability by causing genetic mutations in the DNA sequence^10^.

DNA methylation alterations are also associated with drug treatment sensitivity, for example, hypermethylation of DAPK in colon and breast cancer^11^. These findings suggest that aberrations DNA methylation might affect certain pathways that prevent cancer cells from advancing towards apoptosis or other cell death-related mechanisms, as well as towards the development of drug resistance.

Although we know that epigenetic and transcriptional mechanisms play an important role in tumor development, there are still gaps in our current knowledge. DNA hypermethylation is specifically and locally augmented at CpG islands of tumor suppressor genes but its role in tumorigenesis is controversial^12^. DNA hypermethylation of tumor suppressor genes or genes involved in cell cycle processes are more frequent than their mutation in cancer cells. Consequently, we observe hundreds of methylated DNA regions in cancer cell lines, whereas we only find a few mutated genes that drive tumor onset. Different genes and DNA methylation regions play a role in different types of cancers, and therefore it is even harder to get a clear view of the interplay between DNA methylation and gene expression in carcinogenesis. Identifying key characteristic profiles of DNA methylated regions and alterations in gene expression in cancer cell lines is therefore of major relevance for understanding epigenome/transcriptome interactions in human tumors.

In the present study, to better understand the interplay between the epigenome and the transcriptome, we propose a systems biology framework that allows us to i) classify samples of cancer cell lines based on their epigenetic and transcriptomic signature, and ii) extract relevant features from these clusters to construct a cross-omics interaction network.

Therefore, we apply a multi-layer Nonnegative Matrix Factorization (multi-layer NMF) to obtain a set of transcriptome/epigenome clusters with their corresponding biological features. Nonnegative matrix factorization has already been successfully applied to distinguish between different types of cancers by extracting relevant genomic features and has been applied to investigate the relationship between omics data^13^. Expanding the workflow with the construction of the genomic interaction networks allows us, to not only study the effect of DNA methylation on one gene but could be used to study how one alternation in that specific gene can influence other genes. This could potentially give new insight into the interplay between epigenetic and transcriptomic alterations in cancer cells.

## Results

**Figure 1:**
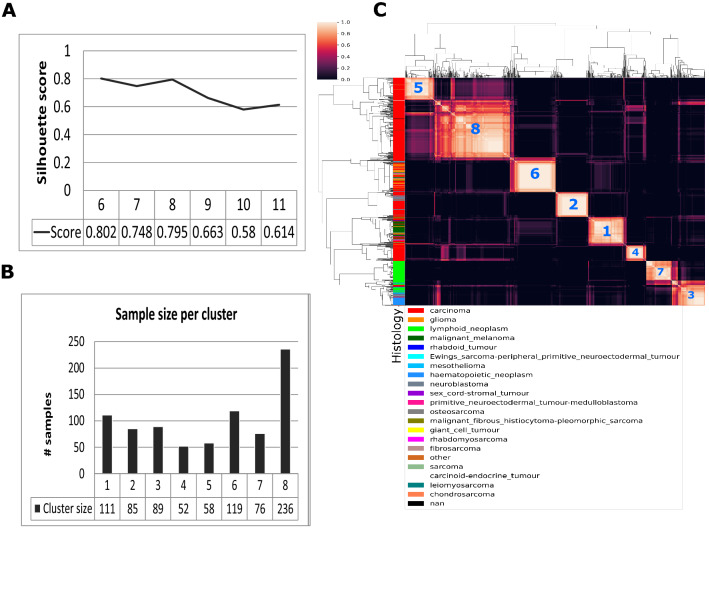
(**A**) Silhouette score for cluster sizes 6 to 11. (**B**) Number of samples in each cluster. (**C**) Consensus plot for cluster size 8.

To estimate the number of clusters in the data set, multiple simulations with different cluster sizes k have been performed to get the silhouette score for every proposed multi-NMF solution. Here, we picked values for *k* in the range of 6 to 11 due to the fact that earlier research suggested at least 6 clusters^14^. Our method predicts the most optimal solution for 8 clusters in our data (Fig. 1A). The solution for k = 8 is above the threshold of 0.7 for a cluster to be regarded stable, but more important, visual inspection of the consensus map of shows multiple stable clusters, as well as a few clusters that contain some noisy samples (Fig. 1C). We observe some clusters that express a strong signal and appear stable across all simulations, while some samples tend to occasionally shift between clusters.

**Figure 2:**
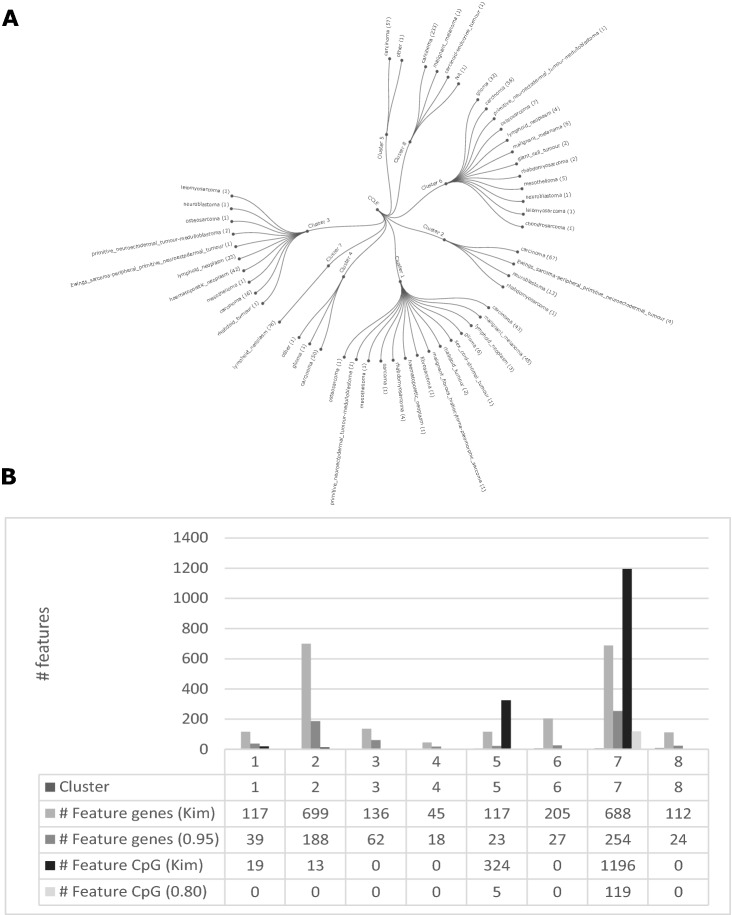
(**A**) The histology of each cluster member as defined by the 2019 CCLE metadata. Here, it becomes apparent that there are a number of clusters with mixed cancer types, but more importantly, there are clusters that show strong homogeneity. (**B**) Histogram for the number of feature genes and DNA promoter regions with Kim score and a more stringent feature scoring.

For each of these clusters, we have identified the number of samples (Fig. 1B), as well as the cancer types of each sample in that particular cluster (Fig. 2A). From Fig. 2A, it becomes clear that we have been able to identify genomic profiles, by the combination of DNA promotor site methylation and gene expression, which results in two homogenous and six heterogeneous clusters. Three clusters (clusters 3, 5, and 8) show a high diversity of cancer types, including carcinomas, sarcomas, and blastomas. However, there are two clusters (cluster 1 and cluster 7) that are very homogenous and consist of carcinomas (cluster 1) and lymphoid neoplasms (cluster 7). These two clusters can be of interest for further investigation, to analyze whether these cancer cell lines consist of a generic DNA promoter methylation and gene expression profile.

For each of the clusters, we extracted unique feature genes and DNA promoter regions that explain the observed clustering (Fig. 2B). Between the clusters, there exists a different weight of the importance of DNA promoter region features versus gene features driving the classification. In clusters 3, 4 and 6 there are no DNA promoter regions found that explained the classification and the cluster is only defined by a set of transcripts. On the contrary, the formation of cluster 7 can be explained by evaluating the combination of the different DNA promoter regions and genes. Here, we have applied two scoring functions: a method by Kim et al.^15^ as introduced in the method section and a more stringent cutoff for features that score > 0.95 for the transcriptome layer and > 0.80 for epigenome layer regions (threshold determined based on the distribution of the scoring functions).

To further investigate the observed heterogeneity within particular clusters, we have looked into various factors that may explain the clustering, including tissue type, TP53 mutation, Race, and Sex (Supplementary File 1). Here, it can be seen that there is a different distribution of tissues over the clusters. Cluster 7 again shows a homogenous distribution of only lymphoid tissue (lymphoid neoplasms), whereas other tissues are distributed across multiple clusters. This may indicate that for those tissue types different genomic profiles are driving the clustering.

**Figure 3:**
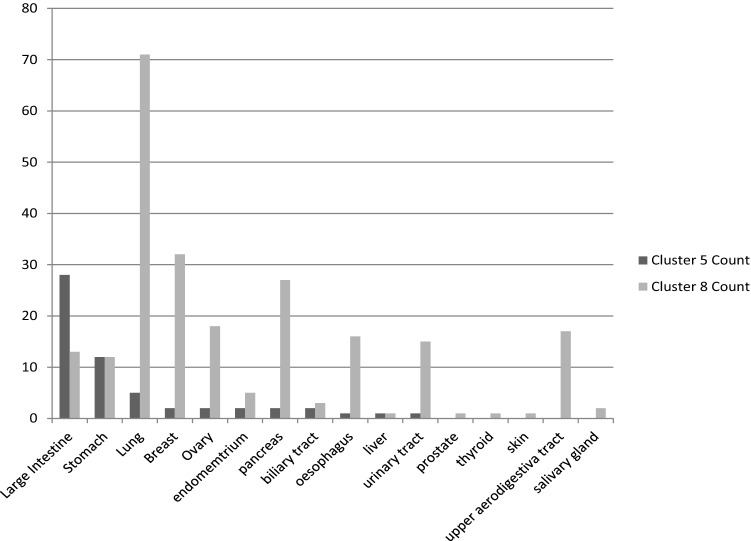
Distribution of the cancer tissues in cluster 5 and cluster 8. All cancer tissues in cluster 5 are also present in cluster 8 but cluster 8 also contains some cancer tissues that are not a member of cluster 5.

**Figure 4:**
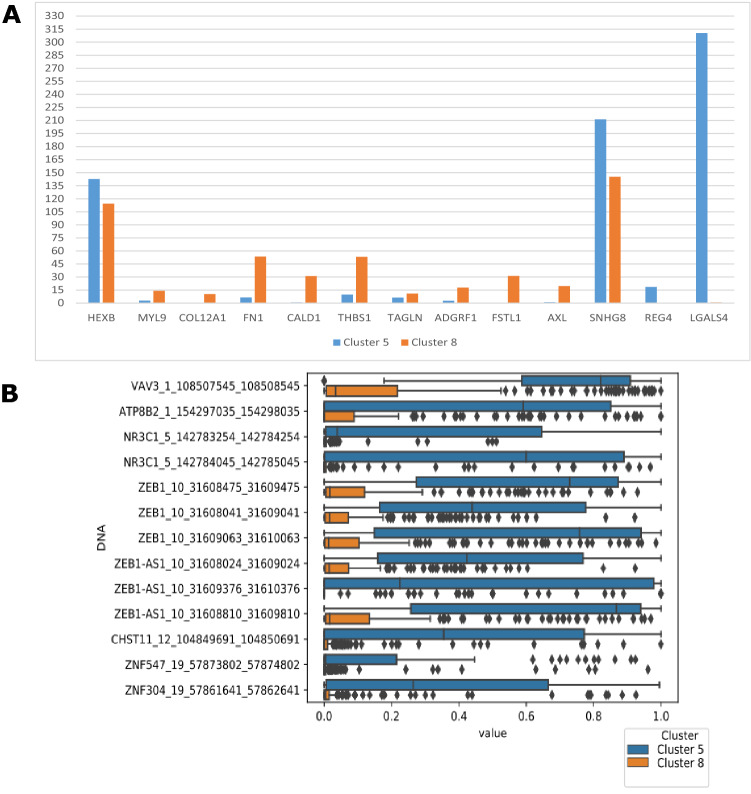
(**A**) Median Log2(TPM + 1) values of genes in cluster 5 and cluster 8. This shows the main drivers behind the stratification of cluster 5 and cluster 8. (**B**) DNA promoter region of cluster 5 and cluster 8. Although cluster 5 and cluster 8 both contain the same cancer tissues, a different methylation pattern is observed for certain regions.

To further investigate why cancer cell lines are separated into different clusters, we have analyzed the features for cluster 5 and 8. Both clusters are selected because of the overlap of the tissue types in cluster 5 in cluster 8 (Fig. 3). This enabled the identification of genomic features that are different between the tissue types (Fig. 4).

A major difference between clusters 5 and 8 is the DNA promoter region methylation of *ZEB1* and *VAV3* (Fig. 4B)*.* A strong difference in methylation patterns across all cell lines is observed between the clusters. Within the transcriptome, a different expression pattern of a set of genes is visible between clusters 5 and 8. For some genes, there is no expression in cluster 5 whereas these are expressed in cluster 8 and vice versa (Fig. 4A, median Log_2_(TPM + 1) value). This includes *FN1*, *CALD1*, *THBS1*, *TAGLN, AXL, HEXB, REG4, and LGALS4*.

Cluster 7 appears to contain a large number of features from both the transcriptome and epigenome platforms. Interestingly, this may point towards a genomic profile that is overlapping between cancer cell lines even though they are histologically different. Therefore, we select this cluster for further investigating the underlying genomic profile.

The features of cluster 7 are mapped against the PantherDB to extract the gene ontology biological processes. Several expressed genes are related to immune response, including the adaptive immune response (p-value 1.28E−09), innate immune response (p-value 2.66E−04) complement activation (p-value 1.44E−02), and immunoglobulin-mediated response (p-value 3.76E−03). For each of the DNA promoter regions, the corresponding gene ID is mapped to identify the biological processes. Here, we found signaling processes such as regulation of signaling (p-value 8.97E−09), negative regulation of signaling (p-value 2.26E−09), regulation of signal transduction (p-value 5.16E−09), regulation of cell communication (p-value 6.97E−08) and Hippo signaling pathway (p-value 1.02E−02).

To identify which genes are specific for blood and lymphoid tissue, we have mapped feature genes to the Human Protein Atlas (HPA) Database^16^, a database that can be used to categorize genes based on expression level and tissue distribution. We have identified 19 enriched genes for blood and lymphoid tissue, as well as genes disease- or cancer-associated genes. For each DNA promoter region, we mapped the associated gene against HPA. Although there are no known lymphoid tissue-enriched genes in the DNA promoter region feature list, there are some known cancer-related genes (see Table 1).

**Table 1:** List of genes and DNA promoter region-associated genes that are either tissue-specific, cancer-related or disease-related.

Blood and lymphoid tissue enriched	Cancer or disease-related	Cancer or disease-related genes
SASH3,CD48	CREB1,FGR	ACVR2A,SPRY2
FGR,SEPT1	TNFSF13B,CD79A	SMAD1,WDFY3
CD86,IGLL5	CD79A,CD19	ITGA2,CRY1
LY9,TNFSF13B	CCR7,CTLA4	PTPRL,LRIG3
CD79A,CD19	TNFSF13,FCRL3	PTK2,BCL2L2
LAX1,VPREB1		NFIB,MYO1E
TNFSF13,IGJ		NRP1,TGIF1
CD28,CCR7		
FCRL3,IGLL1		
FCRL1		

**Figure 5:**
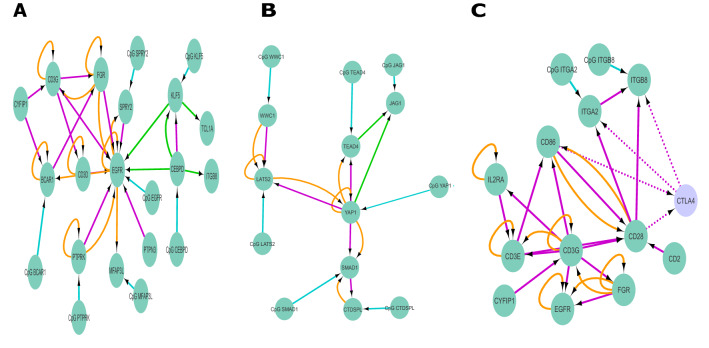
Genomic interaction network modules for cluster 7 (lymphoid neoplasm). (**A**) Subnetwork for the genes connected to EGFR. (**B**) Subnetwork for the region of genes connected to YAP1, TEAD4, JAG1, and SMAD1. (**C**) Subnetwork for the set of genes connected with CD28, CD86, and CTLA4. CTLA4 is a seeding node which is highlighted by the light color and the dotied interactions. Gene–gene interactions are shown in orange, DNA promoter region–gene interactions in cyan, protein–protein interactions in purple, and transcription interactions in green (activation) or red (inhibition).

Finally, we used the DNA promoter regions and expressed genes to construct genomic interactions, to study interactions between and within the transcriptome and epigenome. Here, we focused on the genomic interaction network, because this cluster gave a strong homogeneous signal for lymphoid neoplasms. From the total genomic interaction network (Supplementary Fig. S1), we have identified potential interesting network neighborhoods based on genes that have a high degree, genes that are transcription factors or genes mentioned in Table 1. Figure 5A shows the subnetwork of genes that are located around the epidermal growth factor (*EGFR)*, a gene with a high inner and outer degree. *EGFR* can be transcriptionally activated by two feature methylated genes *KLF5* and *CREBPD.* Furthermore, *EGFR* shares protein–protein interactions with the oncogene *FGR* and *PTK2* and therefore this subnetwork can be important to study in more detail.

The second cluster of genes of interest is located in the neighborhood of *LATS2* and *YAP1* (Fig. 5B). These two genes play a role in the Hippo signaling pathway, a pathway believed to play a pivotal role in cancer^17^. Finally, we have identified a third subnetwork centralized around the lymphoid tissue enriched genes *CD28* and *CD86*, which form the *CD28-CD86* pathway (Fig. 5C). The two subnetworks are of interest because of their role in the signaling pathways.

For each of the genes in the three subnetworks, we compared the gene expression and methylation values for the different feature genes and DNA promoter regions. Here we can see that hypermethylation of the DNA promoter region (Supplementary Fig. S2, plot A–H) corresponds with low gene expression for *EGFR, CEBPD, KLF5, YAP1, LATS2, NFIB, LRCC49,* and *ARHCAP29* (Supplementary Fig. S3: plots A-H)*.*

## Discussion

Cancer is one of the most complex diseases and the same types of tumors can exhibit different genomic traits. The challenge here is to discern whether similar aberrations in different histologies (cross-cancer similarity) have a comparable biological significance. There are five histology classes of cancer: carcinoma, sarcoma, myeloma, leukemia, and lymphoma. Each class has different subclasses according to the origin of the cancer cell. However, there is a shift in the importance of histology as a marker for cancer types. More and more cancers are found to share a set of genetic features, even if they do not belong to the same subclass. It is more important to identify key genomic similarities shared by subgroups of cancer since they present an opportunity to design tumor treatment strategies among tumors regardless of the tissue of origin^18^. Our genomic interaction networks as reported in this study for lymphoid neoplasms could help to identify and further investigate the key genomic characteristics.

Upon having integrated epigenomics and transcriptomics data across a wide range of cancer cell lines, our results demonstrate clusters that contain a mixture of different cancer cell line samples, therefore also a mixture of cancer types. When we look into the genomic features of a given cluster, a set of transcripts and DNA promoter regions is identified that may explain the separation of the same cancer tissues. In cluster 5, which contains the same type of cancer tissues as cluster 8, a different methylation profile is observed in the DNA promoter regions of some key genes. This is of great interest since this might point towards the fact that the same cancer tissues have different epigenetic and transcriptomic alterations.

There are different transcripts and methylated DNA promoter regions that explain the clustering of the different cancer tissues in cluster 5 or 8. One major difference is the role of certain DNA promoter regions in cluster 5, whereas there is no methylation effect predicted to play a role in cluster 8 (Results in Fig. 4B). If we take into account only the DNA promoter regions with a high probability of explaining cluster 5, it is appears that *ZEB1, ZEB1-AS,* and *VAV3* are the most important genes that are hypermethylated. Highly expressed *ZEB1* is associated with malignancy of various cancers, and it plays an important role in cancer transformation^19^. *VAV3* is involved in cell signaling and tumorigenesis^20^ and is a prognostic factor of poor prognosis in breast cancer patients^21^ as well as an important driver of prostate cancer^22^. The hypermethylation of both *ZEB1* and *VAV3* might indicate that those genes do not play a role in the development and progression of the different cancer cell lines in cluster 5.

Besides the different methylation features, several gene transcripts explain the differences between clusters 5 and 8. In cluster 5, a member of the regenerating gene (REG) family members, *REG4,* is predicted to be discriminative transcriptomic features. The REG family members are small secreted lectin-like proteins involved in hepatic, pancreatic, gastric, and intestinal cell proliferation and differentiation^23^. Aberrant expression of *REG4* is associated with tumor growth, survival, adhesion but also resistance to apoptosis^23^. Elevated expression of *FN1* cluster 8 is of interest, because *FN1* is an important gene involved in the development of various cancer types driving proliferation^24,25^. *AXL* expression is associated with various processes in cancer, including proliferation, survival, metastasis and resistance to cancer therapy^26^. Due to the role of *AXL* it has been proposed as target for cancer therapy^26,27^. Due to absent expression of *AXL* in cluster 5, it might not be an effective strategy for those cancer cell lines. *LGALS4* is a protein-coding gene for the protein galectin 4. Galectins are associated with various diseases including cancer and regulate tumor cell adhesion and migration^28^. Moreover, galectin 4 serves as a strong prediction for metastatic potential of adenocarcinomas^29^, a type of carcinoma.

Although heterogeneity seems to play an important role in the clustering of the cancer cell lines, there is one cluster that shows homogeneity towards a class of cancers. Cluster 7 shows a strong intensity for lymphoid neoplasms. This cluster could give us more insight into the underlying epigenetic and transcriptomic changes in lymphoid neoplasms.

The samples in cluster 7 are from a group of disorders that originate from the neoplastic transformation of lymphocytes. Normally, lymphoid stem cells develop into lymphoid blasts that differentiate towards B or T lymphocytes. Recent research has shown that chronic lymphocytic leukemia and multiple myeloma have a shared biological basis^30^. Furthermore, follicular lymphomas and diffuse large B cell lymphomas show shared gene expression patterns associated with immune escape mechanisms^31^. These current insights show that B and T cell lymphomas potentially share genomic alterations. Lymphoma and leukemia originate from white blood cells, thus potentially share the same genomic alterations leading to the development of normal white blood cells towards cancer cells. Therefore it is of interest to deeper investigate this genomic interaction network.

It is of no surprise that pathways analysis of the nodes in the genomic interaction network shows several genes involved in immune response regulation. It is known that lymphoid neoplasms is a disease associated with immunological ignorance and immune evasion^32^.

In the genomic interaction network for cluster 7, various nodes can be identified that are of potential interest. Here we have made a selection based on methylation status, gene expression, and the role of a specific gene in the established genomic interaction network (Supplementary Fig. S1). Methylated promotor regions of *NFIB, ARHGAP29,* and *LRRC49* are predicted to be a feature of cluster 7 meaning that there are drivers of cluster formation. For most samples in cluster 7, the promoter region of *NFIB*, *ARGHAP29,* and *LRRC49* is hypermethylated. In these samples, the genes *NFIB* and *LRRC49* both have low expression values, whereas *ARHGAP29* is not expressed at all. *ARHGAP29* is one of the protein-coding genes for Rap1 that regulates Rho GTPase signaling. Dysregulation of Rap1 activation is responsible for the development of malignancy^33^. Furthermore, RAP1 interacts with many members of the DNA damage response pathway but RAP1-depleted cells show reduced interaction between DNA ligase IV and DNA-pk and are impaired in DNA ligase IV recruitment to enable efficient repair of damaged chromatin^34^.

*NFIB*, with an increased DNA promoter methylation in cluster 7 cell lines, is a transcription factor regulating the maturation of megakaryocytes, a platelet precursor^35^. Megakaryopoiesis is the developmental process of bone marrow progenitor cells into mature megakaryocytes and is required for normal hemostasis. From the genomic interaction network, we can identify possible interactions between *NFIB* and other genes. *NFIB* shares genetic interactions with *FGR* and *CD28*. FGR is a proto-oncogene of the Src family of tyrosine kinases expressed in immune cells^36^. Src family kinases are most of all best known for their role in tumor development and progression^37^. *FGR* is not only connected to *NFIB*, but *FGR also* shares a protein–protein interaction with *IGLL5* and a gene–gene interaction with *FCRL1. FCRL1* expressed in a majority of chronic lymphocytic leukemia, follicular lymphoma, hairy cell leukemia, and mantle cell lymphoma^38^ and might play an important role in the onset of these malignancies. It is therefore of interest to investigate whether the hypermethylation of *NFIB* can be reversed and whether *NFIB* is capable of downregulating *FCRL1* via genetic interactions with FGR.

One of the central nodes in the network is *EGFR*, a gene responsible for controlling cellular proliferation, apoptosis, angiogenesis, and metastatic spread in a variety of cell types and tissues^39^. In cluster 7, it is evident that *EGFR* is hypermethylated and consequently is not expressed (expression level of 0 TPM). Because of the hypermethylation of the promoter region, the transcription factors *CEBPD* and *KLF5* cannot transcriptionally activate *EGFR* expression (Fig. 5A). Even if the DNA promoter region of *EGFR* would be hypomethylated, transcription activation of *EGFR* might not occur, since both *CEBPD* and *KLF5* are not expressed in the cancer cell lines of cluster 7. The combination of hypermethylation of *EGFR* and the inactivity of *CEBPD* and *KLF5* is interesting since *EGFR* shares different gene–gene and protein–protein interactions with *FGR*, *CD3D, CD3G, BCAR, PTK2,* and *PTPN3.* The inactivity of *EGFR* could be of importance since this may alter the interactions with *FGR* and *PTK2* and potentially disrupt the functioning of these oncogenes*. EGFR* expression is still a subject of debate in leukemia^40^ but in lymphomas, it has been demonstrated to increase drug resistance^41^. Our results show low expression of *EGFR* which could potentially mean that *EGFR* cannot contribute to drug resistance and highlight the mechanism of low *EGFR* expression in these cancer cell lines.

A second local neighborhood of interest is defined around *YAP1*, a gene believed to be involved in the regulation of the hematopoietic system^42^. The role of YAP is important, since in solid tumors it emerges as an oncogene, whereas YAP seems to exert a tumor-suppressive function in multiple myeloma and leukemia^42^. In our network, *YAP1* can regulate the transcription of *JAG1* and might interact with *LATS2*, *TEAD4,* and *SMAD1* via protein–protein and gene–gene interactions (Fig. 5B). These possible interactions and transcriptional activation might be altered because of the methylation status of *YAP1*, which shows a trend towards a higher methylated DNA promoter region, and as a possible effect, there is no *YAP1* expression observed in the cancer cell lines in cluster 7. This result is in agreement with previous research, where downregulation or deletion of *YAP1* in multiple myeloma and leukemia is reported^43^. Due to the inactivity of *YAP1*, it will be of interest to determine whether JAG1 and *TEAD4* are expressed*. TEAD4* is low expressed in the cell lines of cluster 7, which could be favorable since *TEAD4* expression is associated with tumor onset and progression^44^. *JAG1* is involved in the NOTCH signaling pathway and downregulation of *JAG1* has been proposed as a target for treatment, since *JAG1* can function as an oncogene in the different lymphoid neoplasms^45^. Similar to *TEAD4*, *JAG1* is lowly expressed in cluster 7, which could be because *YAP1* is not expressed and therefore cannot activate *JAG1* transcription. Although *YAP1* is proposed as a potential tumor suppressor gene^42^, increasing *YAP1* expression might lead to transcriptional activation of the oncogene *JAG1* (Fig. 5B). In our genomic interaction network, there is also an interaction between *LATS2* and *YAP1*. This interaction is actually of interest since *LATS2* and *YAP1* are two genes involved in the hippo signaling pathway^17^. As mentioned before, *YAP1* has a low gene expression due to DNA hypermethylation and therefore we believe that this protein–protein interaction is affected. Furthermore, *LATS2* is low expressed in cluster 7 in comparison with the other clusters, which could be a consequence of the increased methylation of the DNA promoter region of *LATS2.* This could indicate that in cluster 7 the hippo-signaling pathway might not function properly because of DNA hypermethylation and low gene expression of both *LATS2* and *YAP1.*

A third region of interest emerged while studying the local neighborhood of the genes *CD28, CD86, CD80, ITGA2, and CTLA4. CD28* and *CD86* are both lymphoid tissue enriched genes^16^. The two genes form a co-stimulatory pair and upon *CD86-*activation*, CD28* can carry out different functions involved in the Th1 differentiation pathway^46^, cytokine production, and downstream signaling events of the B cell receptor through the activation of *NFkB*^47^*.* In the gene interaction network with *CD28* and *CD86*, the dotted interactions around the seeding node *CTLA4* are of relevance (Fig. 5C). As a seeding node, *CTLA4* does not belong to the features for cluster 7 but its absence is of interest. It becomes clear that *CTLA4* is not expressed in any of the cancer cell lines, whereas *CD28* and *CD86* are expressed only in cluster 7 (Supplementary Fig. S3: plot I–K). *CTLA4* is an inhibitor of the *CD28–CD86* activation pathway and humans that carry any *CTLA4* mutations are found to suffer from profound autoimmunity^48^. *CD86* shows elevated expression in cluster 7 in comparison to the other clusters, which could not only indicate that *CD86* is specific for lymphoid neoplasms, but also that the signaling pathway of *CD86-CD28* is perturbed leading to *CD28* stimulation. The inactivity of *CTLA4* might result in a loss of the inhibition of the signaling pathway of *CD86-CD28* which impacts the differentiation of blood cells (Th1 and B cells) but more interesting, the association of *CTLA4* with autoimmunity might point towards a hypothesis that lymphoid neoplasms might share the same alterations as autoimmune diseases^49^. The absence of *CTLA4* might have other implications, due to the protein–protein interactions with *ITGB8* and *ITGA2*. Dysregulation of the *CD28–CD86* pathway could propagate to *EGFR* and *FGR expression* via the different *CD3* genes as shown in the network.

The previously discussed features are of interest because their changes in expression do not occur for all lymphoid neoplasms. The lymphoid neoplasm samples in clusters 3 and 6 do not have an increased expression of both *CD28* and *CD86* (Supplementary Fig. S3). Cluster 6 shows increased *LRRC49* expression whereas this gene is low expressed in all lymphoid neoplasms samples in cluster 7. Furthermore, the hypermethylated DNA regions in cluster 7 are hypomethylated in cluster 3 and cluster 6 (Supplementary Fig. S2). The combination of the epigenetic and transcriptomic changes stratify the lymphoid neoplasms in different clusters and might therefore be of relevance.

By integration of the omics layers employing Multi-layer Nonnegative Matrix Factorization, we are capable of separating clusters based on their DNA methylation and gene expression profiles across a wide range of cell lines derived from multiple human cancer types. The combination of these profiles leads to heterogeneous clusters of sarcomas and carcinomas, but also more homogeneous clusters of lymphoid neoplasms. Although our method can extract signals that characterize different cancer types, there is still room for improvement. Heterogeneity remains a problem, which will be difficult to solve. One way to overcome this is by performing omics integration on one class of cancer cell lines. We expect that this would improve the integration and would select more subtype-specific signals. However, our findings from the complete 2019 CCLE clarify that our method is indeed capable of identifying possible important characteristics. We can identify different methylated DNA promoter regions in the same cancer tissues, but we are also able to construct a genomic interaction network for lymphoid neoplasms based on specific genomic features for that cancer type. This genomic interaction network helps us to identify the possible relationship between methylated genes and other genes in the network. We have identified different methylated DNA promoter regions that affect transcriptional activation of *EGFR, which* might impact on the protein–protein interactions with the oncogenes *FGR* and *PTK2*. The DNA hypermethylation of *EGFR* could be of interest since this gene contributes to drug resistance. We showed that hypermethylation of *YAP1* leads to low gene expression and as a consequence no transcriptional activation of JAG1. Although *YAP1* has tumor-suppressive characteristics, it is relevant to take into account that this may lead to transcriptional activation of the oncogene *JAG1.* Finally, through the genomic interaction network, we could identify a potential dysregulation of the CD28-CD86-CTLA4 axis in the different lymphoid neoplasms cancer cell lines.

## Conclusion

Characterizing the epigenome and transcriptome is vital for our understanding of cancer cell line behavior, not only for better understanding the cancer-related processes but also for future treatment and anti-cancer drug developments. Here, we have identified potential candidate genes that characterize cancer cell lines of the type for lymphoid neoplasms. Our current insights show that, although assumed different, B and T cell lymphomas potentially share similar genomic alterations. These key alterations are important to study and further understand the development and progression of lymphoid neoplasms.

## Method

### Gene expression and DNA methylation data

For this study, normalized gene expression data and DNA promoter methylation data have been downloaded from the Cancer Dependency Portal (DepMap). Gene expression data is downloaded as Log_2_(TPM + 1) expression values. Gene expression levels have been measured through RNA-sequencing on the Illumina HiSeq 2000 or HiSeq 2500 instruments with sequence coverage of no less than 100 million paired 101 nucleotides-long reads per sample. RNA-seq reads were aligned to the GrCH37 using STAR 2.4^14^.

DNA methylation is measured by Reduced Representation Bisulfite Sequencing (RRBS) analysis to assess promoter methylation. RBBS utilized the MspI cutting pattern to digest DNA to enrich for CpG dinucleotides^50^. The fragments are sequenced on an Illumina HiSeq 2000 and aligned to the hg19 genome using MAQ^50^. A fixed window size of 1000 bp upstream of the transcription starting site for each gene is used to calculate a coverage-weighted average of CpG methylation. RRBS yielded robust coverage of 17,182 gene promoter regions with average coverage greater than 5 reads for the 843 cell lines.

### Multi-layer nonnegative matrix factorization

The original data matrix *X*_i_ is estimated by the product *HW*_*i*_ for each data layer (Eq. 1). To find a local optimal solution, matrices *W*_*i*_ and *H* are updated by their update rules (Eqs. 2 and 3 respectively) and minimizing the Kullback–Leibler divergence (Eq. 4). For *H* we take into account the effect of the different omics layers via *X*_*i*_ and *W*_*i*_ whereas for *W*_*i*_ we take into the effect the omics layers via *X*_*i*_ and the sample clustering via *H*. In the end, *n* matrices *W* are obtained that store the latent features and one coefficient matrix *H* that stores the clustering coefficients.

1 $$ \mathop \sum \limits_{i = 1}^{n } X_{i} \approx \mathop \sum \limits_{i = 1}^{n} W_{i} H $$

2 $$ W_{w + 1} = W*\frac{{\frac{{X_{i} }}{{W_{i} H}}H^{T} }}{\sum H} $$

3 $$ H_{H + 1} = H*\frac{{\sum \frac{{X_{i} }}{{W_{iH} }}^{T} }}{{\sum W_{i}^{T} }} $$

4 $$ KL \;divergence = \sum \left( {X_{i} *\log \left( {\frac{{X_{i} }}{{W_{i} H}} - X_{i} + W_{i} H} \right)} \right) $$

### Feature extraction from NMF results

To analyze the difference in methylation and gene expression profile of each cluster, each matrix W_i_ is scored by using the method proposed by Kim et al.^15^. For each cluster, the entities are selected as features, if those entities that have a high probability of explaining a cluster.

### Genomic interaction networks

The biological features obtained for each cluster are used to create a genomic interaction network. These networks consist of DNA promoter region–Gene interactions, to study the relationship between DNA methylation and gene expression, to identify transcription factor–target interactions, Gene–Gene interactions, and protein–protein interactions as well as to gather information about cell line-specific genes related to cancer. In the genomic interaction network, we allow connections if both genes are in the feature list or if expressed genes from the feature list are connected by one seeding node.

Gene–Gene interactions have been downloaded from OmniPathDb^51^ for each in the extracted feature list. Transcription factor–target interactions are added to the network from a transcription factor library built by Souza et al.^52^, while protein–protein interactions have been downloaded from StringDB^53^.

### Tissue or cancer specific genes and CpG regions

The Human Protein Atlas (HPA) database is used to download information on tissue specificity for lymphoid tissue. From the HPA data we have selected those genes classified as enriched. Genes are categorized enriched when their normalized expression levels are four times higher in a tissue of interest compared to all other tissues. Cancer or disease specific genes are identified if there is evidence that their protein form is disease or cancer related.

## Supplementary Information

Supplementary Figure 1.

Supplementary Figure 2.

Supplementary Figure 3.

Supplementary Information 1.

## Data Availability

The data that support the findings of this study are openly available in the Dependency Map portal at https://depmap.org/portal , reference number^14^.
