# Supplementary material for: From multi-omics integration towards novel genomic interaction networks to identify key cancer cell line characteristics
Source: Sci Rep. 2021 May 18;11:10542. doi: 10.1038/s41598-021-90047-3 (PMC8131752; doi:10.1038/s41598-021-90047-3)

**Supplementary Figure 2**

**From multi-omics integration towards novel genomic interaction networks to identify key cancer cell line characteristics**

T.J.M. Kuijpers^1,^*, J.C.S. Kleinjans^1^ and D.G.J. Jennen^1^

^1^ Department of Toxicogenomics, GROW School for Oncology and Developmental Biology, Maastricht University, P.O. Box 616, 6200 MD, Maastricht, the Netherlands

Email: [tim.kuijpers@maastrichtuniversity.nl](mailto:tim.kuijpers@maastrichtuniversity.nl), [danyel.jennen@maastrichtuniversity.nl](mailto:danyel.jennen@maastrichtuniversity.nl), [j.kleinjans@maastrichtuniversity.nl](mailto:j.kleinjans@maastrichtuniversity.nl)


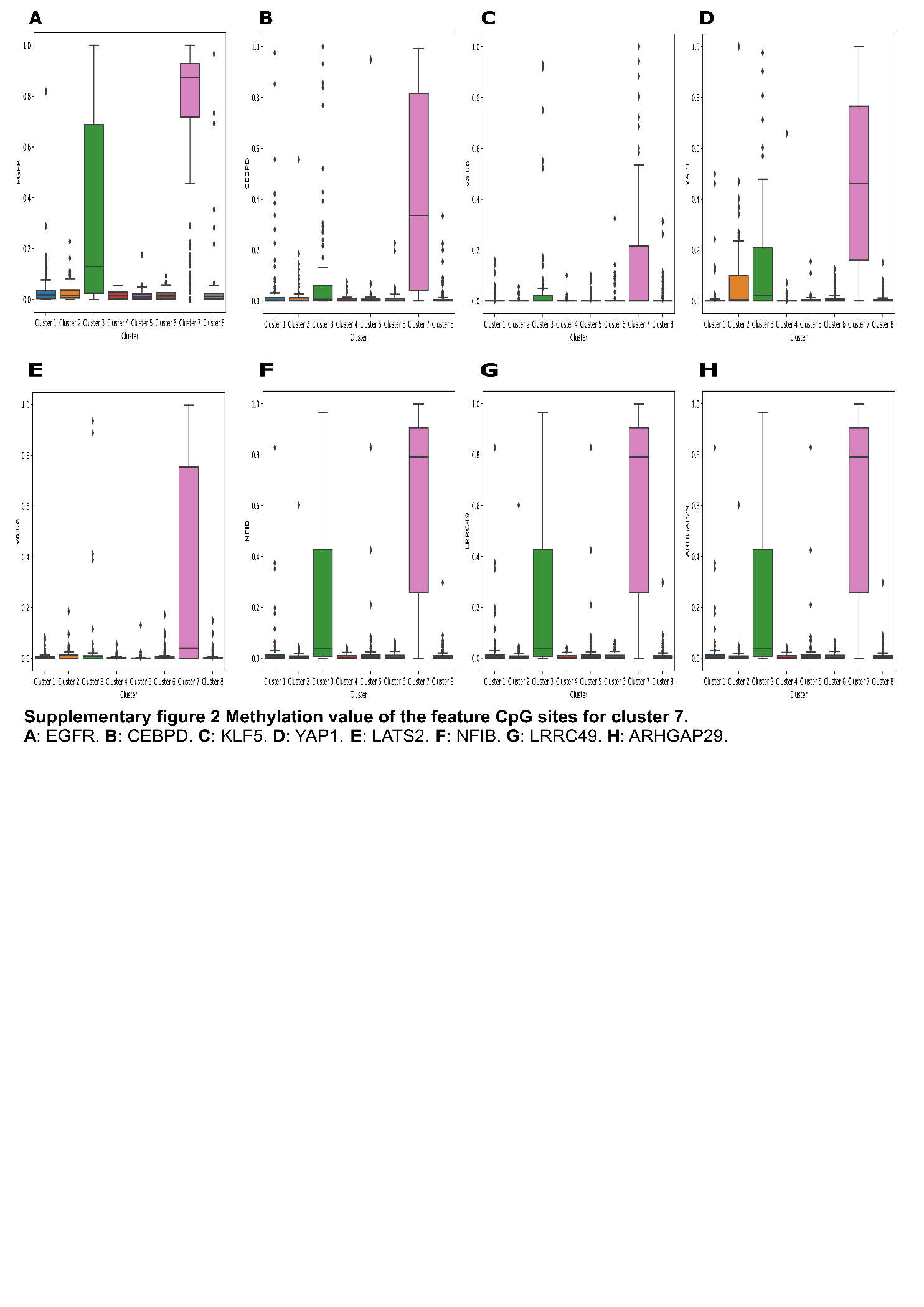

Supplement: Supplementary file 2 — Supplementary Figure 2. [file 41598_2021_90047_MOESM2_ESM.docx]
